# Comparison of the Phenolic Profiles of Soaked and Germinated Peanut Cultivars via UPLC-QTOF-MS

**DOI:** 10.3390/antiox8020047

**Published:** 2019-02-20

**Authors:** Qiong-Qiong Yang, Lin Cheng, Zhi-Yuan Long, Hua-Bin Li, Anil Gunaratne, Ren-You Gan, Harold Corke

**Affiliations:** 1 Department of Food Science & Technology, School of Agriculture and Biology, Shanghai Jiao Tong University, Shanghai 200240, China; yangqiongqiong@sjtu.edu.cn (Q.-Q.Y.); crystalminibee@sjtu.edu.cn (L.C.); 2 Department of Resources & Environment, School of Agriculture and Biology, Shanghai Jiao Tong University, Shanghai 200240, China; qq598892422@sjtu.edu.cn; 3 Guangdong Provincial Key Laboratory of Food, Nutrition and Health, Guangdong Engineering Technology Research Center of Nutrition Translation, Department of Nutrition, School of Public Health, Sun Yat-Sen University, Guangzhou 510080, China; lihuabin@mail.sysu.edu.cn; 4 Faculty of Agricultural Sciences, Sabaragamuwa University of Sri Lanka, Belihuloya P.O. Box 02, Sri Lanka; anil@agri.sab.ac.lk

**Keywords:** groundnut, germination, polyphenols, flavonoids, mass analysis

## Abstract

Diverse peanut varieties are widely cultivated in China. However, few studies have investigated the effects of germination on the phenolic profiles and antioxidant activities of specific Chinese peanut cultivars. Therefore, this study was conducted to evaluate the effects of germination on total phenolic content (TPC), total flavonoid content (TFC), antioxidant activity, and phenolic profiles of seven peanut cultivars in China. The TPC, TFC, and antioxidant activities were determined by spectrophotometry, while phenolic profiles were analyzed by using ultra-high performance liquid chromatography coupled with quadrupole/time-of-flight mass spectrometry (UPLC-QTOF-MS). The results found that germination significantly increased TPC, TFC, and antioxidant activity. Antioxidant activity was found to be closely related to TPC in germinated peanut extracts, which indicates that phenolics are the main contributors of antioxidants in germinated peanuts. In addition, germination induced significant changes in polyphenolic profiles. In the analyzed samples, 36 phenolic compounds were identified in which most were flavonoids. Overall, these findings highlight that germinated peanuts can be a good natural source of natural antioxidants for human consumption and functional food development.

## 1. Introduction

Germination of edible seeds typically involves *de novo* synthesis of bioactive compounds in which many are considered health-enhancing ingredients [1]. Many edible seeds, such as lentils (*Lens culinaris* L.), kidney bean (*Phaseolus vulgaris* L.), mung bean (*Phaseolus aureus* L.), faba bean (*Vicia faba* L.), lupin (*Lupinus angustifolius* L.), and soybean (*Glycine max*), have been germinated to obtain sprouts with improved nutritional and medicinal values [2,3,4,5]. It has been demonstrated that germination can significantly accumulate diverse bioactive compounds such as polyphenols, gamma-aminobutyric acid, and vitamins, and enhance antioxidant capability in germinated seeds [1]. As a result, germinated seeds have received greater attention due to their better health benefits when compared to seeds [6].

The peanut (*Arachis hypogaea* L.) or groundnut is a multi-purpose oil seed widely cultivated in China with many health benefits. Peanut seeds and germinated peanuts are nutritious and have high medicinal and economic values [7]. It has been reported that germinated peanut contains higher levels of flavonoids and other phenolic compounds than peanut seeds [8]. Especially, compared to peanut seeds, germinated peanuts exhibit enhanced antioxidant activity [8]. These phenolic compounds have been associated with a wide range of health benefits such as the prevention of coronary heart diseases, different types of cancer, diabetes, and obesity [9]. Given the health benefits and commercial value, it is useful to comprehensively analyze the phenolic profiles and bioactivities of germinated peanuts.

The evaluations of phenolic profiles and antioxidant activity of local peanut seeds were performed in Canada [10], Japan [11], Argentina [12], and many other countries. Variability of bioactive components and their antioxidant activities in peanut skin extracts have been found due to different growing conditions and cultivars [10,11,12]. Different peanut cultivars have also been widely cultivated in China and the planting area is increasing annually. However, little research has been done on the effects of germination on the phenolic profiles and antioxidant activities of peanut cultivars in China.

Therefore, the purpose of this study was to evaluate the effects of germination on phenolic profiles and antioxidant activities of seven peanut cultivars grown in China. We hope that germination can increase phenolic content and produce some new antioxidant compounds in germinated peanuts. The results of this research can provide valuable information for germinated peanuts as nutraceuticals or functional foods.

## 2. Materials and Methods 

### 2.1. Preparation of Soaked and Germinated Peanuts

Seven peanut cultivars were purchased from online shops of the Taobao Mall. Peanuts were soaked and germinated, according to a previous study [13] with minor modification. After rinsing three times with deionized water, the peanuts were soaked in deionized water (1:10, *w*/*v*) for 10 h at room temperature (22 ± 1 °C). The soaked peanuts were drained, rinsed three times with deionized water, and then germinated in a domestic semi-automatic germination machine (Model CB-A323B, Connie, Guangdong, China) for 10 days in the dark. During the germination process, the temperature was automatically controlled at about 25 °C, and the peanuts were automatically watered every 10 min with fresh deionized water. After germination, germinated peanuts (Day 10) and corresponding soaked peanuts (Day 0) were manually peeled, lyophilized, ground into a fine powder, and stored at 4 °C for further analysis. Germination was performed in triplicate.

### 2.2. Extraction Procedure

According to the previous study and considering its green chemistry property, 70% of ethanol was selected as the extraction solvent [14]. Each sample (0.5 g) was extracted with 10 mL 70% ethanol in a shaker (130 rpm) for 24 h at 50 °C. The extract was then centrifuged at 3000× *g* for 15 min at room temperature (22 ± 1 °C), the supernatant was collected and stored at −20 °C, and it was then analyzed within one week.

### 2.3. Determination of Total Phenolic and Flavonoid Contents

The total phenolic content (TPC) was determined by the Folin-Ciocalteu method, as previously described [15], and the results were expressed as milligrams of gallic acid equivalent (mg GAE)/100 g DW of samples. Total flavonoid content (TFC) was determined by the AlCl_3_-based colorimetric method, as previously described [8], and the results were expressed as mg catechin equivalent (CE mg)/100 g dry weight (DW) of samples.

### 2.4. Determination of Free Radical Scavenging Activity by 1,1-Diphenyl-2-Picrylhydrazyl (DPPH) Method

The DPPH assay was carried out as previously described [16] with minor modification. The DPPH solution was prepared by dissolving DPPH in 80% methanol to obtain an absorbance of 0.70 ± 0.05 at 515 nm. The reaction was conducted by mixing 3.9 mL of DPPH solution with 100 μL of the sample and then reacted at room temperature in dark for 2 h. After that, a UV-Vis spectrophotometer (UV1800PC, Jinghua Technology Instrument Co., Ltd. Shanghai, China) was used to record the absorbance of the solution at 515 nm. A Trolox external calibration curve was established (*y* = 0.5085*x* − 0.009, *R*^2^ = 0.9988), and the results were expressed as mmol Trolox/g DW.

### 2.5. Ferric Ion Reducing Antioxidant Power (FRAP) Assay

The FRAP assay was carried out as previously described [16] with some modifications. Fresh FRAP reagent was prepared by adding 300 mM sodium acetate buffer, 10 mM 2,4,6-Tris(2-pyridyl)-s-triazine (TPTZ) solution, and 20 mM FeCl_3_ solution in the ratio of 10:1:1, and then warmed at 37 °C. The test was conducted by adding 100 μL of sample solution to 3 mL of FRAP reagent, and then reacted for 4 min. After that, a UV-Vis spectrophotometer was used to record the absorbance of the solution at 593 nm. A FeSO_4_ solution external calibration curve was established (*y* = 0.45*x* + 0.00993, *R*^2^ = 0.9984), and the results were expressed as mmol Fe (II)/g DW.

### 2.6. UPLC-QTOF-MS Analysis

A Primer ultra-high performance liquid chromatography coupled with quadrupole/time-of-flight mass spectrometry (UPLC-QTOF-MS) (Waters, Milford, MA, USA) equipped with an electrospray ionization source was used to analysis the phenolic profile of soaked and germinated peanut extracts. Samples were separated on a BEH C18 column (2.1 mm × 100 mm, 1.7 μm) (Waters, Milford, MA, USA) with the following settings: column temperature, 45 °C, flow rate, 0.4 mL/min, mobile phase A, ultrapure water containing 0.1% (*v*/*v*) formic acid, mobile phase B, acetonitrile containing 0.1% (*v*/*v*) formic acid, and injection volume, 1 μL. The gradient conditions were: 0 min, 5% B, 3 min, 20% B, 10 min, 100% B. Mass spectrometric analysis was performed in a negative ion mode with the spectrometer settings as follows: capillary, 2 kV, sample cone voltage, 40 V; desolvation gas temperature, 450 V, flow rate of desolvation gas, 900 L/h, flow rate of cone gas, 50 L/h, source temperature, 115 °C, acquisition range, 50–1000 *m*/*z*, scan rate, 0.2 s, collision energy, and 6 eV/20–45 eV. To ensure accuracy and reproducibility, all analyses were conducted using an independent reference spray via the Lock Spray interface with the setting as follows: lock mass, Tyr-Gly-Gly-Phe-Leu (leucine-encephalin, 250 pg/μL), flow rate, 10 μL/min, interval, 0.5 s, sample time, 0.5 s, and collision energy, 6 eV. The mass data were processed with MassLynx 4.1 software (Waters, Milford, MA, USA). The exact elemental composition of each parent and generated fragments were calculated with a molecular formula calculator. All compounds were identified or tentatively identified by comparing the retention time, parent ion, and mass fragments with those in references and the mass spectrometry database. 

### 2.7. Statistical Analysis

All the measurements were performed in triplicate and the results were expressed as mean ± standard deviation (SD), with a *p*-value less than 0.05 defined as statistically significant. The statistical software GraphPad Prism (Graphpad Software, Inc., San Diego, CA, USA) was used for statistical analysis. 

## 3. Results and Discussion

### 3.1. Changes of TPC and TFC Induced by Germination

**Figure 1:**
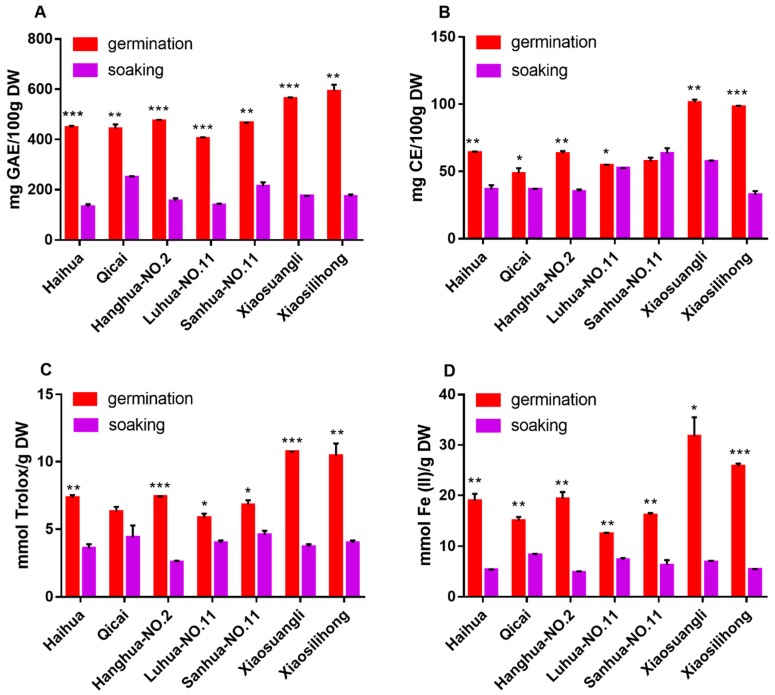
Evaluation of antioxidant phenolics in soaked and germinated peanut extracts. (**A**) Total phenolic content. (**B**) Total flavonoid content. (**C**) DPPH free radical scavenging activity. (**D**) Ferric-reducing antioxidant power. * *p* < 0.05, ** *p* < 0.01, *** *p* < 0.001. GAE: gallic acid equivalent; DW: dry weight; CE: catechin equivalent.

The changes occurring in germinated peanut extracts were investigated with respect to their TPC and TFC values (Figure 1). TPC in soaked peanuts varied from 132.5 to 248.8 mg GAE/100 g DW in all tested cultivars, among which the soaked seeds of Qicai cultivar had the highest TPC value of 248.8 mg GAE/100 g DW (Figure 1A). The TPC observed here were higher than those in Korean-grown peanuts [8], which may be due to different extraction conditions, growth conditions, and genotypes. On the other hand, the TPC in germinated peanuts ranged from 403.4 to 596.2 mg GAE/100 g DW, which was significantly higher than that of the soaked seeds. This indicates that germination could significantly accumulate phenolic compounds. The germinated peanuts from Xiaosilihong and Xiaosuangli cultivars had the highest TPC value among all the tested cultivars, which is even higher than that of most germinated edible seeds and sprouts [1].

The TFC in soaked peanut seeds ranged from 36.6 to 63.4 mg CE/100 g DW with the highest TFC value in peanut seeds of Xiaosuangli cultivar (Figure 1B), which was less than that of the peanut skins [17]. The TFC in germinated peanuts were 48.6–101.3 mg CE/100 g DW. This was significantly higher than that in soaked seeds. However, the TFC in germinated peanuts of Sanhua-NO.11 cultivar was lower than that in soaked seeds. Previous publications on TFC of germinated peanuts were very limited, but it was reported that germinated peanuts contained 63.0 to 730.8 μg quercetin equivalent per gram samples [8]. Generally, germination can significantly accumulate TPC and TFC, which is in agreement with the previous conclusion [1,18]. 

### 3.2. Changes of Antioxidant Activities Induced by Germination

It has been widely accepted that the antioxidant ability should be evaluated by at least two assays because the experimental results obtained in different assays may be inconsistent [19]. In this study, antioxidant activity was evaluated by a DPPH free radical scavenging assay and a ferric-reducing antioxidant power (FRAP) assay. The results are shown in Figure 1C,D.

**Figure 2:**
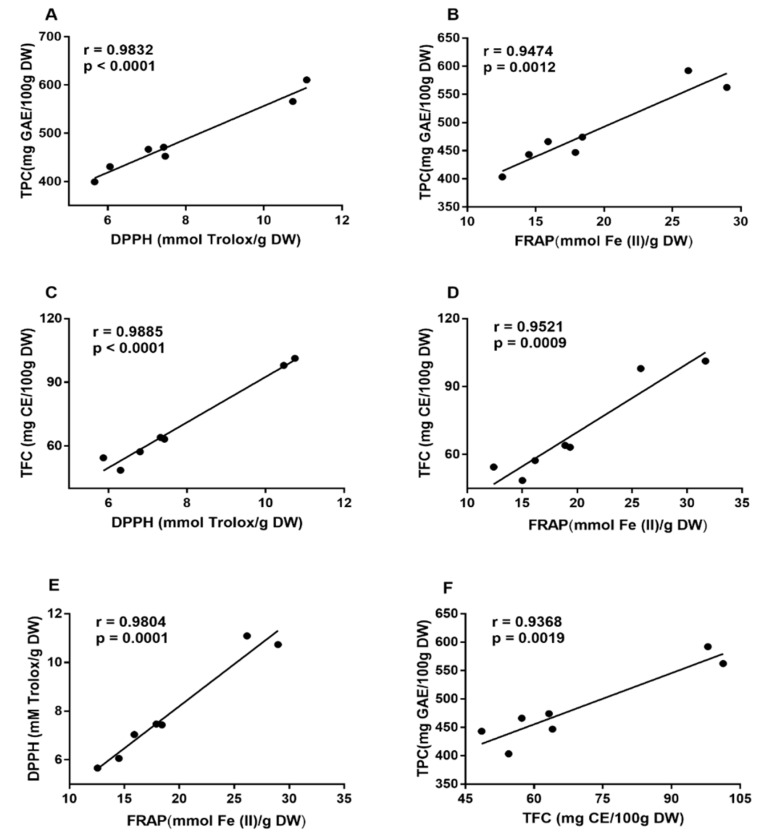
Correlation analysis. (**A**) Correlation between total phenolic content (TPC) and 1,1-diphenyl-2-picrylhydrazyl (DPPH) of germinated peanuts. (**B**) Correlation between TPC and ferric ion reducing antioxidant power (FRAP) of germinated peanuts. (**C**) Correlation between total flavonoid content (TFC) and DPPH of germinated peanuts. (**D**) Correlation between TFC and FRAP of germinated peanuts. (**E**) Correlation between DPPH and FRAP of germinated peanuts. (**F**) Correlation between TPC and TFC of germinated peanuts. GAE: gallic acid equivalent; DW: dry weight; CE: catechin equivalent.

The DPPH and FRAP values of soaked peanut extracts, respectively, ranged from 2.65 to 4.80 mmol Trolox/g DW and 4.98 to 8.09 mmol Fe (II)/g DW. Soaked peanut extracts of Qicai cultivar exhibited the strongest antioxidant activity measured by the two assays. Despite its relatively strong antioxidant capability, it is still less effective than germinated peanut extracts (Figure 1C,D), which showed DPPH values ranged from 5.66 to 11.09 mmol Trolox/g DW and FRAP values ranged from 12.56 to 28.98 mmol Fe (II)/g DW. All of them were significantly higher than the corresponding soaked peanuts, which indicates that germination can significantly enhance antioxidant capacity of peanuts. In addition, in both assays, germinated peanut extracts of Xiaosilihong and Xiaosuangli cultivars had the highest antioxidant activity. We speculate that this phenomenon is related to phenolic contents because it has been demonstrated that there is a positive correlation between antioxidant capacity and TPC in fruits [20], vegetables [21], flowers [22], red rice [23], spices [24], and medicinal plants [25]. Therefore, we further confirmed this conclusion by generating the correlation coefficients of TPC, TFC, DPPH, and FRAP data. From Figure 2, we can see that TPC had a high correlation coefficient with the DPPH value (*r* = 0.9832, *p* < 0.0001) and FRAP value (*r* = 0.9474, *p* = 0.0012), which indicates that the antioxidant capacity of germinated peanuts might be mainly attributed to phenolics in germinated peanuts. Additionally, TFC also had a positive correlation with the DPPH value (*r* = 0.9885, *p* < 0.0001) and the FRAP value (*r* = 0.9521, *p* = 0.0009), which further demonstrated that the antioxidant capacity was closely related to flavonoid compounds in germinated peanuts. There was a positive correlation between DPPH and FRAP values (*r* = 0.9804, *p* = 0.0001), which indicates that antioxidants in germinated peanuts can not only scavenge free radicals, but also reduce oxidants. These results also confirmed that both antioxidant assays are consistent with each other. In addition, TPC and TFC also exhibited positive correlation (*r* = 0.9368, *p* = 0.0019), which suggests that flavonoids can be the main phenolics in germinated peanuts.

### 3.3. Characterization of the Phenolic Profile of Peanut Extracts by UPLC-QTOF/MS

**Figure 3:**
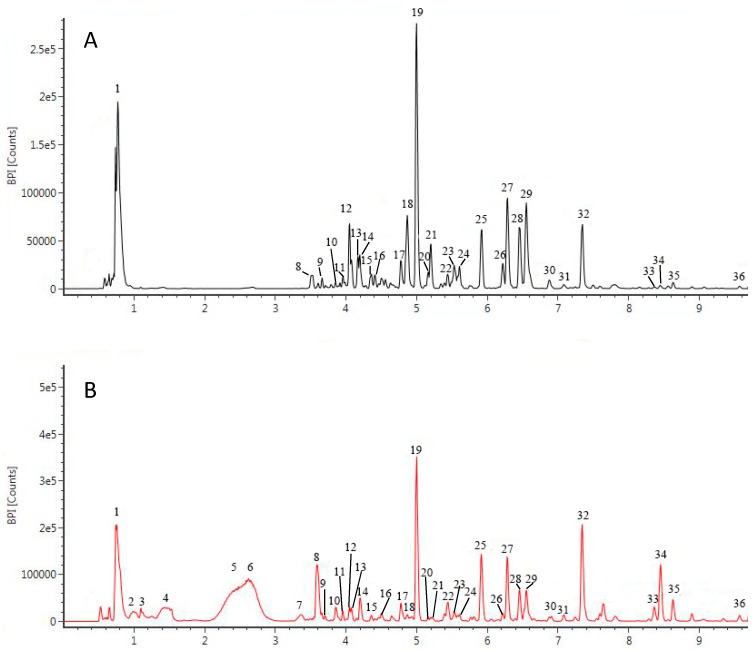
The base peak ion chromatograms (BPI) of soaked (**A**) and germinated (**B**) peanut extracts. Numbers indicate peaks of compounds found in extracts.

**Figure 4:**
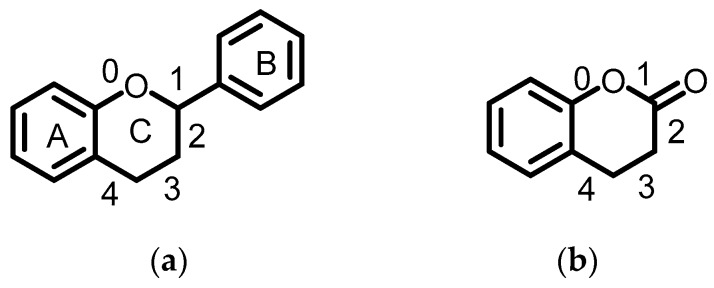
The backbones of flavonoids (**a**) and coumarins (**b**), and their potential cleavage bones marked with numbers. A, ring A; B, ring B; C, ring C.

According to Figure 1A, germinated Xiaosilihong extracts had relatively higher TPC. In order to find out the changes of the phenolic profile induced by germination, we further analyzed the phenolic profile of soaked and germinated Xiaosilihong extracts by UPLC-QTOF/MS. Corresponding base peak ion chromatograms (BPI) were shown in Figure 3. All tentatively identified compounds were elucidated by their retention time (min), molecular ions, fragment ions, calculated molecular formula of each peak, and by matching the above information with relevant references and internal mass spectrometry database. Notably, we have tentatively identified 36 compounds, including three flavanols, four flavanones, three flavones, eleven flavonols, one isoflavonoid, one anthocyanin, four coumarins, and nine others. Previous studies found that many phenolic compounds mainly exist in the peanut skins [26,27,28,29]. However, our study demonstrates that they can also exist in soaked and germinated peanut extracts without skins. The backbones of flavonoids and coumarins are shown in Figure 4, and their potential cleavage bonds are marked with numbers.

#### 3.3.1. Flavanols

Peak 7 was tentatively identified as kushenol X due to its molecular ion [M + HCOO]^−^ at *m*/*z* 485.1792 and fragments at *m*/*z* 381.13543 [M–H_2_O–allyl]^−^, 317.13501 [M–ring B–CH_3_]^−^, 267.09830 [^1,3^A–H_2_O]^−^, 257.11352 [^1,4^A]^−^, 243.09824 [^1,4^A–CH_3_]^−^, 229.08526 [^0,3^A–allyl]^−^. Peak 10 was tentatively identified as catechin-O-hexose because it gave a molecular ion [M–H]^−^ at 451.1244 and a mass spectrometry (MS^2^) signal at *m*/*z* 289.07164, which is consistent with the loss of hexose, thus releases the catechin moiety [30]. Other fragment ions can be obtained through proper cleavage pathways including m/z 341.08758 [M–ring B]^−^, *m*/*z* 179.03466 [M–ring B-glucopyranosyl]^−^, and *m*/*z* 137.06046 [^1,3^B–H_2_O]^−^. Peak 32 had a molecular ion [M–H]^−^ at 441.0826, which was associated with deprotonated catechin-O-gallate [31], and fragment ions at *m*/*z* 163.03966 [M–ring B–O-galloyl]^−^, *m*/*z* 149.06054 [^1,4^B–O-galloyl]^−^, *m*/*z* 131.04992 [^1,4^B–O-galloyl–H_2_O]^−^, *m*/*z* 121.02923 [^0,3^A]^−^, and *m*/*z* 93.03425 [ring B–H_2_O]^−^.

#### 3.3.2. Flavanones

Peak 17 had a molecular ion at 523.1456, which was tentatively identified as epiafzelechin-3-O-(6”-O-acetyl)-allosepyranoside, according to its fragment ions at *m*/*z* 461.10846 [M–CH_3_]^−^, *m*/*z* 443.10154 [M–CH_3_–H_2_O]^−^, *m*/*z* 435.12843 [M–acetyl]^−^, *m*/*z* 371.13447 [^0,4^B]^−^, 357.11921 [^0,2^A]^−^, *m*/*z* 327.10736 [M–ring B–O-acetyl]^−^, *m*/*z* 321.09529 [^1,3^B–H_2_O]^−^, *m*/*z* 315.1086 [^0,2^A–acetyl]^−^, *m*/*z* 299.11352 [^1,3^B–acetyl]^−^, *m*/*z* 281.10318 [^1,3^B–O-acetyl]^−^, *m*/*z* 233.06661 [^2,4^B–CH_3_]^−^, *m*/*z* 181.05017 [M–ring B–allosepyranosyl]^−^, *m*/*z* 137.02418 [^1,3^A]^−^, and *m*/*z* 135.04472 [^1,3^B–allosepyranosyl]^−^. Peak 21 was tentatively identified as naringenin-4′-glucoside-7-rutinoside with a molecular ion [M–H]^−^ at 741.2263, according to its fragment ions and mass spectrometry database. The fragment ion at *m*/*z* 473.13097 was obtained by losing ring B and a methyl group from its parental ion, while the *m*/*z* 325.09298 was obtained by losing a rhamnosyl and ring B. Other fragment ions including *m*/*z* 445.13519 [^1,3^A–H_2_O]^−^, *m*/*z* 433.13527 [^1,3^A], *m*/*z* 299.07755 [^1,3^A–O- rhamnosyl]^−^, and *m*/*z* 137.02411 [^0,3^A–rutinosyl]^−^, could further confirm its structure. Peak 25 was tentatively identified as narirutin with a molecular ion [M–H]^−^ at 579.1729, which was also detected by He et al. [32], and fragment ions at *m*/*z* 458.10399 [^1,3^A]^−^, *m*/*z* 289.07185 [M–ring B–O-mannopyranosyl–2H_2_O]^−^, *m*/*z* 283.04641 [^1,4^A–mannopyranosyl]^−^, and *m*/*z* 162.05562 [O-mannopyranosyl]^−^. Peak 31 was tentatively identified as eriocitrin with a molecular ion [M–H]^−^ at 595.1671, which can lose ring B and two molecular water giving *m*/*z* 437.14277, or can lose rutinosyl obtaining *m*/*z* 285.03977. Other fragment ions at *m*/*z* 300.08585 [^1,3^A–O-mannopyranosyl]^−^, *m*/*z* 161.04521 [O-mannopyranosyl]^−^, *m*/*z* 152.019098 [^1,3^A-rutinosyl]^−^, and *m*/*z* 135.04415 [^1,3^B]^−^ could further confirm that it was eriocitrin, which was also detected in *Citrus aurantium* L. var. amara Engl. [33].

#### 3.3.3. Flavones 

Peak 20 was tentatively identified as isoetin-O-hexose-O-xylose because its molecular ion [M–H]^−^ at 595.1304 and a fragment ion at *m*/*z* 300.02742, consistent with the loss of hexose and xylose, released isoetin moiety [34]. Other fragment ions further confirmed its structure including *m*/*z* 463.08836 [M- xyloypyranosyl]^−^, *m*/*z* 271.02475 [^1,4^B–H_2_O–H_2_O]^−^, and *m*/*z* 243.03003 [^1,3^B–H_2_O–H_2_O]^−^. Based on the above molecular ion and fragment ions, peak 20 was tentatively identified as isoetin-O-hexose-O-xylose. Peak 26 was tentatively identified as apigenin-O-rhamnose-6’’-O-acetyl–glucoside, according to its molecular ion [M–H]^−^ at 619.1643 and fragment ions. The molecular ion could lose an acetyl group giving m/z 577.15336, or lose ring B, O-acetyl, and two molecules of water giving *m*/*z* 435.12702, or lose ring B, acetyl, one molecule of water, and O-rhamnosyl giving *m*/*z* 300.02716. Other potential cleavage pathways further confirmed the potential structure of the compound, such as *m*/*z* 457.13497 [^1,4^A–H_2_O]^−^, *m*/*z* 325.09243 [^1,3^A–H_2_O–O-rhamnosyl]^−^, *m*/*z* 281.10271 [^0,4^A–H_2_O–O-rhamnosyl]^−^, *m*/*z* 163.03943 [^0,4^B]^−^, *m*/*z* 145.02891 [^1,4^B]^−^, and *m*/*z* 119.04974 [^1,3^B]^−^. Peak 28 was identified as isoscoparin-3’’-O-glucopyranoside with a molecular ion [M–H]^−^ at 623.1614, which further lost ring B and two molecules of water give *m*/*z* 463.08797. The fragment ion at *m*/*z* 299.05591 corresponding to [M–3-O-glucopyranosylglucopyranoside]^−^ and the fragment ion at *m*/*z* 285.04021 [M–3-O-glucopyranosylglucopyranosid–CH_3_]^−^, which further lost one molecule of water and gave *m*/*z* 269.04569. Fragment ion at *m*/*z* 306.09794 corresponded to [3-O-glucopyranosylglucopyranoside–H_2_O]^−^, and the fragment at *m*/*z* 301.03039 was obtained by losing ring B, O-glucopyranosyl, and one molecule of water from a molecular ion. This type of flavone was also reported in a previously published paper [35].

#### 3.3.4. Flavonols

Peak 9 was tentatively identified as dehydrosilybin with molecular ion [M–H]^−^ at 479.0985 and a fragment at *m*/*z* 432.08485 [M–H_2_O–CH_2_OH]^−^. The *m*/*z* 325.10441 was obtained by losing two molecules of water from [^1,4^B]^−^, while *m*/*z* 271.09352 was obtained by losing one molecule water from ring B, which further lost hydroxymethyl obtaining *m*/*z* 255.06350. The fragment at *m/z* 153.01842 corresponded to [^1,3^A]^−^ of benzopyran. The compound also had fragment ions at *m*/*z* 123.04478 corresponding to the molecular ion of 2-methoxyl-phenyl, which further lost one methyl obtaining *m*/*z* 109.02871. Peak 15 and 19 had a molecular ion [M + HCOO]^−^ at 785.2145, which was tentatively identified as kaempferol-3-O-(2G-α-L-rhamnosyl)-rutinose. The fragmented ion at *m*/*z* 597.18020 corresponding to loss of ring B and three molecules of water, *m*/*z* 472.18237 was [3-O-(2G-α-L-rhamnosyl)-rutinosyl]^−^ and the fragment ion at *m*/*z* 443.15701 was [^1,3^B–rhamnosyl]^−^. The *m*/*z* 161.4529 corresponds to [O-rutinosyl]^−^. Other fragment ions can be obtained through proper cleavage pathways including *m*/*z* 542.10944 [M- O-rhamnosyl–methyl–H_2_O]^−^, *m*/*z* 395.04263 [M–CH_2_-O-rhamnosyl–rhamnosyl]^−^, and *m*/*z* 325.05632 [M–CH_2_-O-rhamnosyl–rhamnosyl–ring B]^−^. Peak 16 was tentatively identified as isorhamnetin-O- gentiobioside with a molecular ion [M–H]^−^ at 639.1667. The molecular ion lost a methyl moiety, which gave a fragment ion at *m*/*z* 623.12088 or lost a glucopyranosyl obtaining *m*/*z* 477.10398. The fragment ion at *m*/*z* 465.10444 was obtained by losing ring B and three molecular water. Fragment ion *m*/*z* 315.05086 was obtained by losing gentiobiosyl and releasing isorhamnetin [36], which further lost a methyl giving *m*/*z* 300.02680. While the *m*/*z* 178.02665 was obtained by losing ring B and O-gentiobiosyl from the molecular ion. Peak 23 was tentatively identified as kaempferol-3-getiobioside with a molecular ion [M–H]^−^ at 609.1458 and fragment ions at *m*/*z* 283.02432 [M–getiobiosyl]^−^, which was consistent with the molecular ion of keampferol [36], *m*/*z* 273.03861 [^1,4^B–CH_2_-O-glucopyranosyl–H_2_O]^−^, *m*/*z* 243.02971 [^1,3^B–CH_2_-O-glucopyranosyl–H_2_O]^−^, and m/z 151.00330 [^1,3^A]^−^. Peak 24 had a molecular ion [M–H]^−^ at 609.1458, which was tentatively assigned as kaempferol-di-O-hexoside. The fragment ion at *m*/*z* 300.02716 was obtained by losing ring B, one molecular glucopyranosyl, and two molecular water from the parental ion, while the *m*/*z* 283.04641(keampferol [36]) was obtained by losing two molecular glucopyranosyl from the parental ion. Other fragment ions were obtained by the cleavage of bonds of ring C, including *m*/*z* 273.03921 [^1,4^B -CH_2_OH–H_2_O]^−^ and *m*/*z* 151.00350 [^1,3^A–glucopyranosyl]^−^. Peak 27 had a molecular ion [M–H]^−^ at 433.0771, which was speculated as quercetin-3-O-xyloside. Its fragment ions were obtained through proper cleavage pathways shown in brackets, with the mass data including *m*/*z* 307.04574 [M–ring B–H_2_O]^−^, *m*/*z* 233.04492 [^1,3^B–CH_2_OH–H_2_O]^−^, *m*/*z* 178.02645 [M–ring B–O-xylosyl]^−^, *m*/*z* 160.01618 [^1,4^B–O-xylosyl]^−^, *m*/*z* 151.03960 [^1,3^B–xylosyl]^−^, *m*/*z* 147.04485 [^1,4^B–O-xylosyl–H_2_O]^−^, and *m*/*z* 93.03419 [ring B–H_2_O]^−^. Peak 29 was tentatively identified as complanatuside with a molecular ion [M–H]^−^ at 623.1616, and fragment ions at *m*/*z* 463.08559 [^0,4^B -2H_2_O]^−^, *m*/*z* 337.09246 [M–ring B–2H_2_O]^−^, which further lost one molecule of water and gave *m*/*z* 315.05056. Other fragment ions, such as *m*/*z* 301.03066 [^1,4^B–H_2_O–glucopyranosyl]^−^, and *m*/*z* 299.05594 [M–glucopyranosyl–glucopyranosyl]^−^, also further confirmed its potential structure [37]. Similar mass data about complanatuside was also reported in *Lepidium draba* L. [38]. Peak 30 had a molecular ion [M–H]^−^ at 477.1035, which further lost O- glucopyranosyl and gave 299.05590, and then lost methyl, which gives *m*/*z* 284.03118, or lost methoxyl, which gives *m*/*z* 268.03702. The molecular ion could further lose glucopyranosyl, which gives *m*/*z* 313.03505, and then loses methyl, which gives *m*/*z* 299.01949. In addition, according to other fragment ions such as *m*/*z* 179.03430 [^0,4^B–glucopyranosyl]^−^, *m*/*z* 178.99793 [^1,3^A]^−^, and *m*/*z* 151.00331 [^1,4^A]^−^, the structure could be speculated as methoxykaempferol-O- hexoside. Peak 33 was tentatively identified as icariin, according to its molecular ion [M + HCOO]^−^ at *m*/*z* 721.2351 and fragment ions. This compound was also reported in a previous study [39]. The fragment ion at *m*/*z* 577.15302 was obtained by losing a methylbut-2-enyl and a hydroxymethyl from the parental ion, while the fragment ion at *m*/*z* 519.14986 was obtained by losing ring B, a hydroxymethyl, and a molecular water from the parental ion. Other fragments could further confirm the structure of icariin and the potential cleavage methods were illustrated in bracket following its corresponding *m*/*z* molecules, including *m*/*z* 499.14498 [M- ring B–H_2_O- allyl]^−^, *m*/*z* 493.11498 [M–methyl–H_2_O- rhamnosyl]^−^, *m*/*z* 383.13546 [^1,3^A]^−^, *m*/*z* 337.09321 [M–ring B–H_2_O- allyl- glucopyranosyl]^−^, *m*/*z* 321.09581 [^1,4^B]^−^, *m*/*z* 284.03285 [M–methyl–glucopyranosyl- rhamnosyl–methylbut-2-enyl]^−^, *m*/*z* 227.07101 [M–ring B–O-glucopyranosyl–O-rhamnosyl]^−^, *m*/*z* 208.03701 [M–ring B–glucopyranosyl–rhamnosyl–allyl]^−^, *m*/*z* 161.04546 [O-rhamnosyl]^−^, and *m*/*z* 145.02909 [^1,4^B–methyl–O-rhamnosyl]^−^. Peak 36 was tentatively identified as heptamethoxyflavone, according to its molecular ion [M–H]^−^ at 431.1319 and fragments ions at *m*/*z* 219.02915 [^0,4^B–methyl]^−^, *m*/*z* 179.03412 [^1,4^A–methoxyl]^−^, and *m*/*z* 177.0496 [^1,4^B–methoxyl–methyl]^−^. Similar flavone derivatives were also reported previously [29].

#### 3.3.5. Isoflavonoids

Peak 35 had a typical molecular ion [M–H]^−^ at 445.1145 and fragment ions at *m*/*z* 287.05591 [^1,4^B–H_2_O]^−^, *m*/*z* 281.10175 [^1,3^B]^−^, *m*/*z* 167.03443 [^1,3^A]^−^, *m*/*z* 117.03423 [^1,3^B–glucopyranosyl]^−^. According to the parental ion, fragment ions, and database, it was tentatively identified as prunetin-4’-glucoside.

#### 3.3.6. Anthocyanins

Peak 18 was identified as malvidin-O-(O-acetyl- glucopyranoside) -O- glucopyranoside with a molecular ion [M + HCOO]^−^ at 741.1884 and fragment ions at *m*/*z* 515.17736 [M–glucopyranosyl–H_2_O]^−^, *m*/*z* 475.08874 [M–acetyl-β-D-glucopyranosyl–CH_3_]^−^, *m*/*z* 343.04591 [M–ring B–glucopyranosyl–2H_2_O]^−^, *m*/*z* 300.02761 [M–ring B–acetyl-glucopyranosyl-2H_2_O]^−^, *m*/*z* 163.03987 [M–ring B–glucopyranosyl–O-acetyl-glucopyranosyl]^−^.

#### 3.3.7. Coumarins 

Peak 8 and 22 were tentatively assigned as dihydrocoumarin isomers due to its typical molecular ion [M–H]^−^ at 147.0449 [40], and fragment ions at *m*/*z* 117.0041, *m*/*z* 103.05505, and *m*/*z* 71.1338 were, respectively, obtained by the cleavage of bonds 2 and 4, bonds 0 and 2, and bonds 0 and 4 from ring B of dihydrocoumarin. Peak 11 and 34 were identified as coumurrayin with a molecular ion [M + HCOO]^−^ at 319.1187 and fragments at *m*/*z* 245.08175 [M–CH_3_–CH_3_]^−^, *m*/*z* 217.5002 [M–2-methyl allyl]-, *m*/*z* 203.10720 [^0,4^A]^−^, *m*/*z* 185.05750 [^1,4^A–methoxyl]^−^, *m*/*z* 171.01148 [M–methoxyl–3-methylbut-2-enyl]^−^, *m*/*z* 165.05497 [^1,4^A–2-methyl allyl]^−^, and *m*/*z* 151.03940 [^1,4^A–3-methylbut-2-enyl]^−^. This compound has also been reported in *Toddalia asiatica* root bark [41].

#### 3.3.8. Others

Peak 1 was identified as protocatechuic acid with a molecular ion [M–H]^−^ at *m*/*z* 153.0189 and fragment ion at *m*/*z* 109.18336 [M–COOH]^−^, which has also been reported in peanut skin extracts [10]. Compared to the profile of soaked peanut extract, peaks 2–6 newly emerged in germinated peanut extract, which potentially contributed to the enhanced antioxidant activity of germinated peanuts. Peak 2 and 4 had the same molecular ion [M–H]^−^ at *m*/*z* 581.1835 and similar fragment ions, and they were tentatively assigned as phenylethyl-trihydroxy-tetrahydro-phenylethyl-hydroxychromone-oxy-chromone. The chromone derivatives were also detected in peanut pods [30]. The fragment ion at *m*/*z* 423.09024 was generated by losing three molecules of water and one molecule phenylethyl from the parental ion, while *m*/*z* 314.04595 was obtained by losing [^1,4^B], one molecule of water, and phenylmethyl from the parental ion. Peak 3 with a molecular ion [M–H]^−^ at *m*/*z* 133.0148. A fragment ion at *m*/*z* 101.02393 [M–H_2_O–H_2_O]^−^ was tentatively identified as 2-hydroxy-succinic acid [42]. Peak 5 was tentatively identified as pantothenic acid with a molecular ion [M + HCOO]^−^ at m/z 292.1397, which lost one molecule of water and 2-methyl propanol and gave *m*/*z* 154.05308. A cleavage of alkyl carbon-nitrogen bond produced a fragment containing nitrogen atom, which further lost one molecule of water and gave a fragment ion at *m*/*z* 130.08695. Similarly, cleavage of the carbonyl carbon-nitrogen bond produced a fragment containing nitrogen atom, which further lost one molecule of water and gave a fragment ion at *m*/*z* 96.04507. Peak 6 was tentatively identified as AH 11 with a molecular ion [M + HCOO]^−^ at *m*/*z* 575.1731 and a fragment ion at *m*/*z* 437.14155 [M–phenyl–H_2_O]^−^. Peak 12 had a fragment ion at 149.04538 corresponding to [O-apiofuranosyl]^−^, a fragment ion at *m*/*z* 161.04525 corresponding to [O-glucopyranosyl]^−^, and a fragment ion at *m*/*z* 137.05051, which was associated with phenylethanol in addition to its molecular ion [M–H]^−^ at *m*/*z* 431.1556. Based on the above analysis, it was tentatively assigned as hydroxyphenylethyl-O-apiofuranosyl-O-glucopyranoside. Peak 13 was tentatively identified as renifolin with a molecular ion [M + HCOO]^−^ at *m*/*z* 397.1505 and fragment ions at *m*/*z* 269.10306 [^0,4^A–H_2_O]^−^, 263.0917 [^1,4^A–H_2_O–H_2_O]^−^, 173.09739 [M–O-glucopyranosyl]^−^, 162.05543 [glucopyranosyl]^−^, and 147.04470 [^1,3^A-glucopyranosyl]^−^. Peak 14 was tentatively identified as tetrahydroxy- hydroxychalcone, according to its molecular ion [M–H]^−^ at m/z 289.0716 and fragment ions at *m*/*z* 163.03974 [M–dihydroxyphenyl–H_2_O]^−^, 119.04989 [M–dihydroxybenzaldehyde–2H_2_O]^−^, 109.02898 [dihydroxyphenyl]^−^, and 93.03428 [hydroxyphenyl]^−^.

## 4. Conclusions

In conclusion, peanut germination induced compositions of phenolic compounds that varied markedly. Meanwhile, the total phenolics and total flavonoids were observed to significantly increase. These variations of phenolic compounds were responsible for the antioxidant activities of germinated peanuts. The positive correlation between phenolic contents and antioxidant activity has profound guiding significance for improving the efficacy of natural antioxidants. In addition, this study found that germinated peanuts of Xiaosilihong and Xiaosuangli cultivars overall had the highest TPC, TFC, and antioxidant activities, which indicates that these two varieties can be selected to produce peanut sprouts rich in antioxidant phenolics.

## References

[1] Gan R.Y., Lui W.Y., Wu K., Chan C.L., Dai S.H., Sui Z.Q., Corke H. (2017). Bioactive compounds and bioactivities of germinated edible seeds and sprouts: An updated review. Trends Food Sci. Technol..

[2] Luo Y.W., Xie W.H. (2013). Effect of germination conditions on phytic acid and polyphenols of faba bean sprouts (*Vicia faba* L.). Legume Res..

[3] Aguilera Y., Liébana R., Herrera T., Rebollo-Hernanz M., Sanchez-Puelles C., Benitez V., Martín-Cabrejas M.A. (2014). Effect of illumination on the content of melatonin, phenolic compounds, and antioxidant activity during germination of lentils (*Lens culinaris* L.) and kidney beans *(Phaseolus vulgaris* L.). J. Agric. Food Chem..

[4] Tajoddin M., Manohar S., Lalitha J. (2014). Effect of soaking and germination on polyphenol content and polyphenol oxidase activity of mung bean (*Phaseolus aureus* L.) cultivars differing in seed color. Int. J. Food Prop..

[5] Lazo-Vélez M.A., Guardado-Félix D., Avilés-González J., Romo-López I., Serna-Saldívar S.O. (2018). Effect of germination with sodium selenite on the isoflavones and cellular antioxidant activity of soybean (*Glycine max*). LWT Food Sci. Technol..

[6] Wang H., Wang J.H., Guo X.B., Brennan C.S., Li T., Fu X., Chen G., Liu R.H. (2016). Effect of germination on lignin biosynthesis, and antioxidant and antiproliferative activities in flaxseed (*Linum usitatissimum* L.). Food Chem..

[7] Akram N.A., Shafiq F., Ashraf M. (2018). Peanut (*Arachis hypogaea* L.): A prospective legume crop to offer multiple health benefits under changing climate. Compr. Rev. Food Sci. Food Saf..

[8] Adhikari B., Dhungana S.K., Ali M.W., Adhikari A., Kim I.D., Shin D.H. (2018). Resveratrol, total phenolic and flavonoid contents, and antioxidant potential of seeds and sprouts of Korean peanuts. Food Sci. Biotechnol..

[9] Bansode R.R., Randolph P., Ahmedna M., Williams L.L., Yu J. (2015). Bioavailability and hypolipidemic effects of peanut skin polyphenols. J. Med. Food.

[10] de Camargo A.C., Regitano-d’Arce M.A.B., Rasera G.B., Canniatti-Brazaca S.G., do Prado-Silva L., Alvarenga V.O., Sant’Ana A.S., Shahidi F. (2017). Phenolic acids and flavonoids of peanut by-products: Antioxidant capacity and antimicrobial effects. Food Chem..

[11] Tsujita T., Shintani T., Sato H. (2014). Preparation and characterization of peanut seed skin polyphenols. Food Chem..

[12] Larrauri M., Zunino M.P., Zygadlo J.A., Grosso N.R., Nepote V. (2016). Chemical characterization and antioxidant properties of fractions separated from extract of peanut skin derived from different industrial processes. Ind. Crops Prod..

[13] Gan R.Y., Wang M.F., Lui W.Y., Wu K., Corke H. (2016). Dynamic changes in phytochemical composition and antioxidant capacity in green and black mung bean (*Vigna radiata*) sprouts. Int. J. Food Sci. Technol..

[14] Miron-Merida V.A., Yanez-Fernandez J., Montanez-Barragan B., Huerta B.E.B. (2019). Valorization of coffee parchment waste (*Coffea arabica*) as a source of caffeine and phenolic compounds in antifungal gellan gum films. LWT Food Sci. Technol..

[15] Gan R.Y., Xu X.R., Song F.L., Kuang L., Li H.B. (2010). Antioxidant activity and total phenolic content of medicinal plants associated with prevention and treatment of cardiovascular and cerebrovascular diseases. J. Med. Plants Res..

[16] Zhu F., Cai Y.Z., Yang X.S., Ke J.X., Corke H. (2010). Anthocyanins, hydroxycinnamic acid derivatives, and antioxidant activity in roots of different Chinese purple-fleshed sweet potato genotypes. J. Agric. Food Chem..

[17] Chuenchom P., Swatsitang P., Senawong T., Jogloy S. (2016). Antioxidant capacity and phenolic content evaluation on peanut skins from 3 peanut types. Chiang Mai J. Sci..

[18] Limmongkon A., Nopprang P., Chaikeandee P., Somboon T., Wongshaya P., Pilaisangsuree V. (2018). LC-MS/MS profiles and interrelationships between the anti-inflammatory activity, total phenolic content and antioxidant potential of Kalasin 2 cultivar peanut sprout crude extract. Food Chem..

[19] Yu L.L., Haley S., Perret J., Harris M., Wilson J., Qian M. (2002). Free radical scavenging properties of wheat extracts. J. Agric. Food Chem..

[20] Fu L., Xu B.T., Xu X.R., Gan R.Y., Zhang Y., Xia E.Q., Li H.B. (2011). Antioxidant capacities and total phenolic contents of 62 fruits. Food Chem..

[21] Deng G.F., Lin X., Xu X.R., Gao L.L., Xie J.F., Li H.B. (2013). Antioxidant capacities and total phenolic contents of 56 vegetables. J. Funct. Foods.

[22] Li A.N., Li S., Li H.B., Xu D.P., Xu X.R., Chen F. (2014). Total phenolic contents and antioxidant capacities of 51 edible and wild flowers. J. Funct. Foods.

[23] Gunaratne A., Wu K., Li D.Q., Bentota A., Corke H., Cai Y.Z. (2013). Antioxidant activity and nutritional quality of traditional red-grained rice varieties containing proanthocyanidins. Food Chem..

[24] Shan B., Cai Y.Z., Sun M., Corke H. (2005). Antioxidant capacity of 26 spice extracts and characterization of their phenolic constituents. J. Agric. Food Chem..

[25] Gan R.Y., Kuang L., Xu X.R., Zhang Y., Xia E.Q., Song F.L., Li H.B. (2010). Screening of natural antioxidants from traditional Chinese medicinal plants associated with treatment of rheumatic disease. Molecules.

[26] Ma Y.Y., Kosinska-Cagnazzo A., Kerr W.L., Amarowicz R., Swanson R.B., Pegg R.B. (2014). Separation and characterization of soluble esterified and glycoside-bound phenolic compounds in dry-blanched peanut skins by liquid chromatography-electrospray ionization mass spectrometry. J. Agric. Food Chem..

[27] Qiu J.Y., Chen L.L., Zhu Q.J., Wang D.J., Wang W.L., Sun X., Liu X.Y., Du F.L. (2012). Screening natural antioxidants in peanut shell using DPPH-HPLC-DAD-TOF/MS methods. Food Chem..

[28] Lopes R.M., Agostini-Costa T.D., Gimenes M.A., Silveira D. (2011). Chemical composition and biological activities of arachis species. J. Agric. Food Chem..

[29] Lee J.H., Baek I.Y., Ha T.J., Choung M.G., Ko J.M., Oh S.K., Kim H.T., Ryu H.W., Park K.Y., Park K.H. (2008). Identification and characterization of phytochemicals from peanut (*Arachis hypogaea* L.) pods. Food Sci. Biotechnol..

[30] Tsamo A.T., Ndibewu P.P., Dakora F.D. (2018). Phytochemical profile of seeds from 21 Bambara groundnut landraces via UPLC-qTOF-MS. Food Res. Int..

[31] Frost S., Lerno L.A., Zweigenbaum J., Heymann H., Ebeler S.E. (2018). Characterization of red wine proanthocyanidins using a putative proanthocyanidin database, amide hydrophilic interaction liquid chromatography (HILIC), and time-of-flight mass spectrometry. Molecules.

[32] He Y.J., Cheng P., Wang W., Yan S., Tang Q., Liu D.B., Xie H.Q. (2018). Rapid investigation and screening of bioactive components in simo decoction via LC-Q-TOF-MS and UF-HPLC-MD methods. Molecules.

[33] Shen C.Y., Jiang J.G., Huang C.L., Zhu W., Zheng C.Y. (2017). Polyphenols from blossoms of *Citrus aurantium* L. var. amara Engl. show significant anti-complement and anti-inflammatory effects. J. Agric. Food Chem..

[34] Shi S.Y., Zhao Y., Zhou H.G., Zhang Y.P., Jiang X.Y., Huang K.L. (2008). Identification of antioxidants from *Taraxacum mongolicum* by high-performance liquid chromatography-diode array detection-radical-scavenging detection-electrospray ionization mass spectrometry and nuclear magnetic resonance experiments. J. Chromatogr. A.

[35] Kim B., Woo S., Kim M.J., Kwon S.W., Lee J., Sung S.H., Koh H.J. (2018). Identification and quantification of flavonoids in yellow grain mutant of rice (*Oryza sativa* L.). Food Chem..

[36] Garcia-Cayuela T., Gomez-Maqueo A., Guajardo-Flores D., Welti-Chanes J., Cano M.P. (2019). Characterization and quantification of individual betalain and phenolic compounds in Mexican and Spanish prickly pear (*Opuntia ficus-indica* L. Mill) tissues: A comparative study. J. Food Comp. Anal..

[37] Yao Y.F., Lin C.Z., Liu F.L., Zhang R.J., Zhang Q.Y., Huang T., Zou Y.S., Wang M.Q., Zhu C.C. (2019). Identification and pharmacokinetic studies on complanatuside and its major metabolites in rats by UHPLC-Q-TOF-MS/MS and LC-MS/MS. Molecules.

[38] Bicha S., Benmekhebi L., Boubekri N., Khellaf R., Brouard I., Zama D., Benayache S., Benayache F. (2016). Compositional study, antibacterial and antioxidant potential of *Lepidium draba* L. (Brascicaceae). Res. J. Pharm. Biol. Chem. Sci..

[39] Sun M.J., Yin Y.W., Wei J., Chen X.P., Ouyang H.Z., Chang Y.X., Gao X.M., He J. (2018). Development and validation of a HPLC-MS/MS method for simultaneous determination of twelve bioactive compounds in epimedium: Application to a pharmacokinetic study in rats. Molecules.

[40] Zheng Y., Xu X.L., Yuan F., Yao M.Y., Ji S.L., Huang Z.Q., Zhang F. (2017). Simultaneous analysis of simple coumarins and furocoumarines in cigarettes by solid-phase extraction with gas chromatography-mass spectrometry. J. AOAC Int..

[41] Zhang X.Y., Sun W.B., Yang Z., Liang Y., Zhou W., Tang L. (2017). Hemostatic chemical constituents from natural medicine *Toddalia asiatica* root bark by LC-ESI Q-TOF MS. Chem. Cent. J..

[42] Rojas-Garbanzo C., Winter J., Montero M.L., Zimmermann B.F., Schieber A. (2019). Characterization of phytochemicals in Costa Rican guava (*Psidium friedrichsthalianum*-Nied.) fruit and stability of main compounds during juice processing–(U)HPLC-DAD-ESI-TQD-MS^n^. J. Food Comp. Anal..

